# Pure Single‐Crystalline Na_1.1_V_3_O_7.9_ Nanobelts as Superior Cathode Materials for Rechargeable Sodium‐Ion Batteries

**DOI:** 10.1002/advs.201400018

**Published:** 2015-02-17

**Authors:** Shuang Yuan, Yong‐Bing Liu, Dan Xu, De‐Long Ma, Sai Wang, Xiao‐Hong Yang, Zhan‐Yi Cao, Xin‐Bo Zhang

**Affiliations:** ^1^ Key Laboratory of Automobile MaterialsMinistry of Education and School of Materials Science and EngineeringJilin UniversityChangchun130012China; ^2^ State Key Laboratory of Rare Earth Resource UtilizationChangchun Institute of Applied ChemistryChinese Academy of SciencesChangchun130022China

**Keywords:** cathode, Na‐ion batteries, Nanobelts, Na_1.1_V_3_O_7.9_, single‐crystalline

## Abstract

**Pure single‐crystalline Na_1.1_V_3_O_7.9_ nanobelts** are successfully synthesized for the first time via a facile yet effective strategy. When used as cathode materials for Na‐ion batteries, the novel nanobelts exhibit excellent electrochemical performance. Given the ease and effectiveness of the synthesis route as well as the very promising electrochemical performance, the results obtained may be extended to other next‐generation cathode materials for Na‐ion batteries.

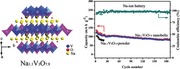

## 1 Introduction

High performance rechargeable batteries are crucial to the development of large‐scale electric energy storage for renewable energies. Following the great success in the field of portable electronics, rechargeable lithium‐ion batteries have risen to prominence as the key energy storage technology for electric vehicle propulsion and in parallel are starting to be evaluated for stationary applications.^1^ However, we shall always be prepared for the exhaustion of limited and unevenly distributed Li resources in the Earth's crust. In response, room‐temperature Na‐ion batteries (NIBs) have again aroused a great deal of interest recently because Na resources are practically inexhaustible and ubiquitous, which is extremely favorable for large‐scale stationary electric energy storage applications for renewable energies and smart grids.^2^, ^3^, ^4^, ^5^ However, partially because Na ion is larger and heavier than Li ion, there is still only very limited number of potential cathode materials for NIBs, and the obtained cathode materials are still far from satisfying in terms of specific capacity, rate capability, and cycling stability.^6^, ^7^, ^8^ Therefore, development of advanced cathode materials for NIBs is urgently desirable but remains a great challenge.

Metal vanadates have been proposed as electrode materials due to their structure flexibility, high discharge capacity, and low cost.^9^ On the other hand, due to their large surface to volume ratio to contact with electrolyte, continuous conducting pathways for electrons through the electrodes, facile strain relaxation during battery operation, etc., 1D structured materials could significantly improve power‐ and energy‐density over bulk electrode materials.^10^ In these context, nanostructure Ag–V–O and Cu–V–O have been successfully synthesized and showed excellent cathode performances in LIBs;[[qv: 9d–9g]] and single crystalline V_2_O_5_ nanobelts indicating a high cathode capacity of 231.4 mAh g^−1^ for NIBs due to their large interlayer spacing.[[qv: 10c]] However, although alkaline metal vanadates (AMVs) have been found to be potential Li^+^ and Na^+^ intercalated electrode materials, there is very few report on AMVs as cathode materials in NIBs, to say nothing of AMVs with uniform morphology, high purity single crystal and 1D structure, which is partially because control of morphology and phase purity of AMVs is very difficult due to their intrinsic phase structure variety and temperature sensitivity. To the best of our knowledge, as a representative example of layered AMVs, the encouraging Na_1.1_V_3_O_7.9_ (NVO) can only be synthesized by complicated or time‐consuming route.[[qv: 11a]],[[qv: 11b]] Even though, the obtained NVO holds irregular shape and inextirpable impurity,[[qv: 11c]] which inevitably casts shadow over its application as cathode materials in rechargeable NIBs. Thereafter, developing new strategy to synthesize pure 1D NVO and then exploring their electrochemical performance toward sodium is of great importance.

Herein, pure single crystalline Na_1.1_V_3_O_7.9_ nanobelts (NVONBs) are successfully synthesized via a facile and low‐temperature strategy using cheap commercial bulk vanadium pentoxide (V_2_O_5_) and sodium hydroxide (NaOH) as V and Na precursor, respectively. The Rietveld refinement method shows that the NVONBs hold layered structure which could facilitate Na^+^ insertion/extraction. Inspired by this structure advantage, when used as cathode materials for NIBs between 1.5 and 3.8 V, the NVONBs exhibit superior electrochemical performances, including high specific capacity of 173 mAh g^−1^, good cycle stability and rate capability, and high coulombic efficiency. Surprisingly, even after 1000 cycles of fast charging/discharging processes, the morphology of NVONBs could keep almost unchanged. Considering the facility and effectiveness of the synthesis route as well as the very promising electrochemical performances, the results obtained here might be extended to other next generation AMVs cathode materials for NIBs.

## 2 Results and Discussion

**Figure 1:**
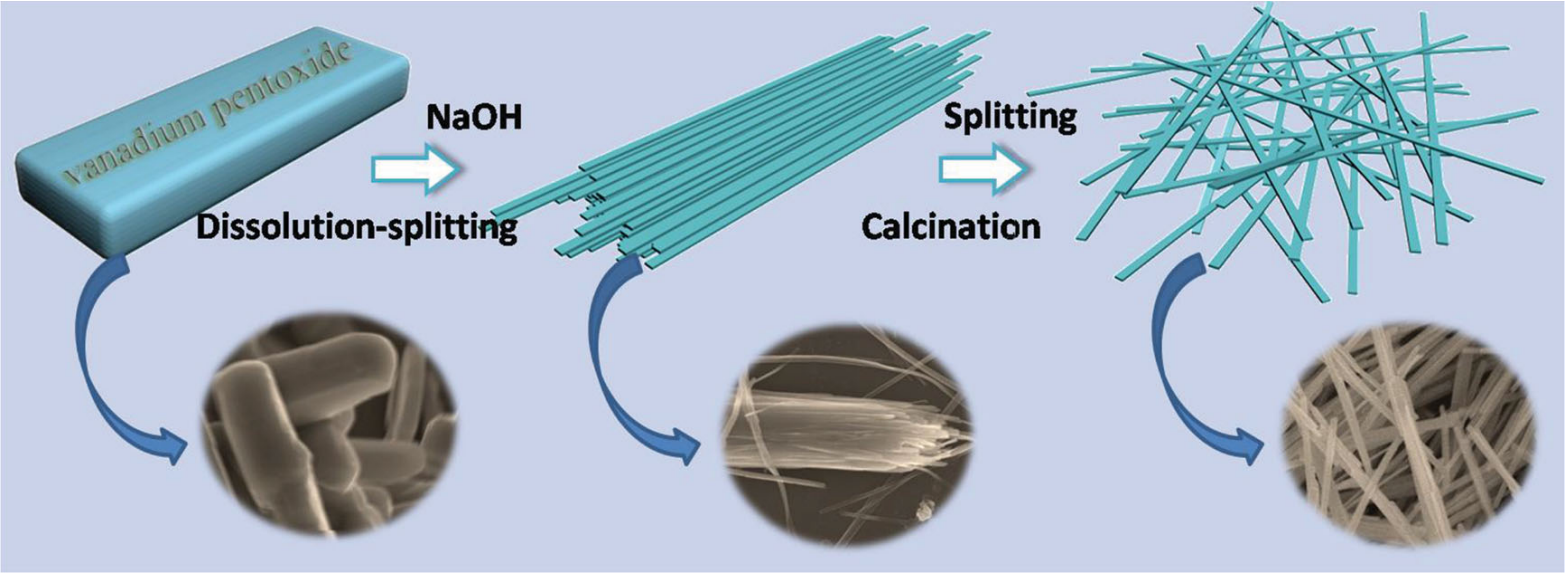
Schematic illustration of the steps for preparation of NVONBs.

**Figure** **1** schematically illustrates the procedure for the preparation of NVONBs. In sodium hydroxide solution, the sodium ions are gradually intercalated into the interspace between the layers of bulk V_2_O_5_ to form intercalated compound Na_2_V_6_O_16_. Meanwhile, partial interlayers of the crystal are dissolved, which leads to the peeling of nanobelts from the parent bulk crystal, that is to say, the splitting process is the dissolving of interlayers. As the reaction proceeds, the internal tension induced a splitting process to lower the total energy, during which the layered Na_2_V_6_O_16_ gradually split into nanobelts. Powder X‐ray diffraction (XRD) pattern and field emission scanning electron microscope (FESEM) image of Na_2_V_6_O_16_ are shown in Figure S1 and S2, Supporting Information, respectively. The obtained Na_2_V_6_O_16_ nanobelts are dried and then calcinated in air to give NVONBs.

**Figure 2:**
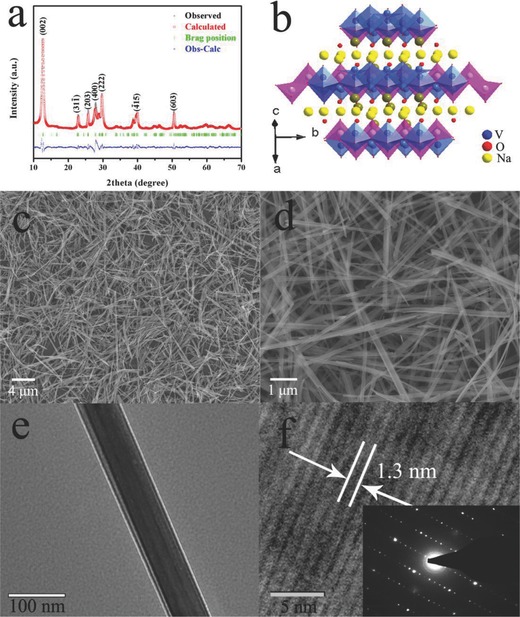
a) XRD patterns of prepared NVONBs and standard NVO. b) Schematic representation of the crystal structure of NVO. c,d) FESEM and e,f) TEM images of NVONBs, inset image is SAED of NVONBs.

The crystal structure and phase purity of the obtained sample are then examined by XRD. **Figure** **2**a shows the Rietveld refinement results of the obtained powder XRD profile. The initial structural model which approximates the actual structure of NVONBs is constructed with crystallographic data previously reported for Na_1.2_V_3_O_8_ structure model. Rietveld refinement confirms that the synthesized NVONBs are well indexed with monoclinic NVO (JCPDS No: 45‐0498, lattice parameters: *a* = 13 Å, *b* = 8.388 Å, and *c* = 14.102 Å, *β* = 101.7°). From the crystal structure depicted in Figure 2b, it can be easily found that the NVONBs hold layered structure with layers of mixed V–O octahedral and tetrahedral units forming slabs parallel to (001), while Na, O atoms are accommodated in the large interlayer space. Pairs of distorted VO_6_ octahedra and distorted VO_5_ tetrahedra oriented parallel to the *b* axis share a common O corner. From such a framework linkage, interstitial channels along *b* axes could provide rather large cavities and the Na^+^ ions in the layer space hold two independent positions. According to the XRD pattern and Rietveld refinement results, the observed reflection can be reasonably assigned to pure NVO. Theoretically, this kind of layered structure could provide open and stable channels for facile Na^+^ ion insertion during discharge and extraction during charge process.[[qv: 10c]],^12^

Morphology of as‐synthesized NVONBs is then characterized using electron microscopy. Low magnification FESEM images (Figure 2c,d) show the NVONBs hold homogeneous nanobelts morphology with size of about 50–150 nm in width and several tens of micrometers in length. Figure 2e,f show transmission electron microscopy (TEM) and high‐resolution TEM images of as‐prepared NVONBs. It is found that the lattice fringe of the layer distance is about 1.3 nm, which is corresponding to the lattice plane of (100) of NVONBs. The microstructure of the NVONBs is further studied by means of the selected‐area electron diffraction (SAED, Figure 2f, inset). Surprisingly, NVONBs with single crystal are found to be successfully synthesized. To the best our knowledge, this is the first time report of single‐crystal NVONBs. It is generally considered that single‐crystal structure materials hold no significant grain boundaries and quite a few defects. Consequently, when it is used for battery electrode, Na/Li‐ion does not need to across grain boundary and defects, which would in principle facilitate Na/Li‐ion diffusion during electrochemical reaction (vide infra).^13^

**Figure 3:**
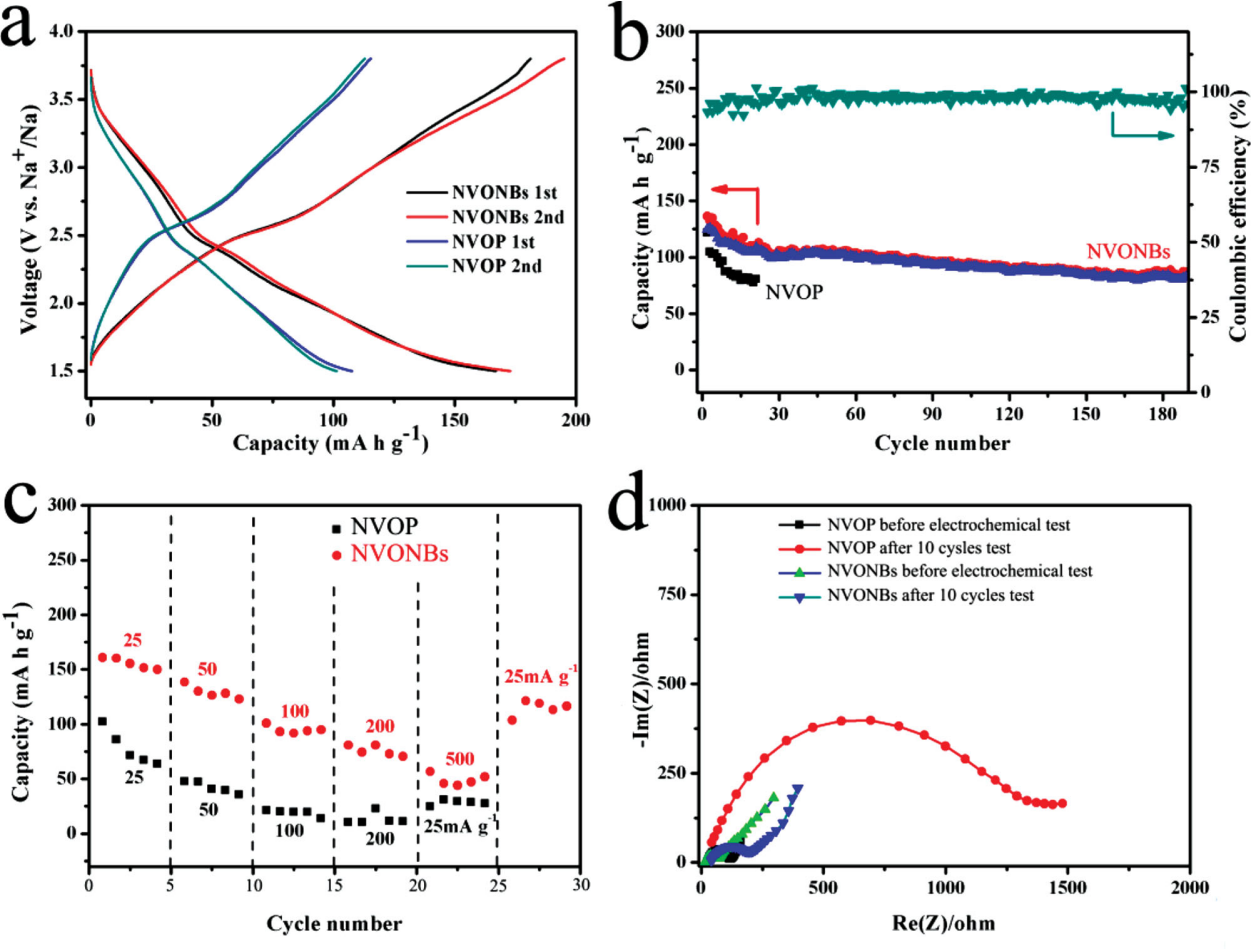
a) Electrochemical performances of NVONBs and NVOP electrodes at a current density of 25 mA g^−1^. b) Cycling performances of NVOP/NVONBs and coulombic efficiency of NVONBs at 50 mA g^−1^. c) Rate capability of NVONBs and NVOP electrodes at various current densities from 25 to 500 mA g^−1^. d) Nyquist plots for NVONBs and NVOP before and after ten cycles.

Inspired by the structure and purity advantage, the electrochemical performance toward sodium of NVONBs is then investigated using coin cells with metallic Na as counter electrode. Galvanostatic charge/discharge technique is used to evaluate the electrochemical performance of NVONBs electrode. For comparison, cycling performance of the irregular‐shaped Na_1.1_V_3_O_7.9_ particles powder (NVOP) (morphology of NVOP is showed in Figure S3, Supporting Information) is also investigated under the same testing conditions. **Figure** **3**a shows the galvanostatic cycling curves obtained for NVONBs and NVOP as cathode electrodes against Na metal at current density of 25 mA g^−1^ between 1.5 and 3.8 V. The irreversible capacity during charge on the first cycle might be caused by the electrolyte decomposition and formation of a solid‐electrolyte interphase (SEI). It should be noted that the sloping charge/discharge profile between 3.5 and 1.5 V might be due to Na^+^ ion insertion into the octahedral sites between the layers composed of vanadium and oxygen atoms, accompanied with some continuous and complex electrochemical reactions between Na, V, and O (vide infra),[[qv: 10c]],^12^, ^13^ which could be potentially solved by introduce some anionic/polyanion to increase the valence state of V to improve the reaction potential, such as F^−^ or PO_4_^−^, etc. After the first cycle, the reactions of both NVONBs and NVOP electrodes show high reversibility as the second cycle can almost well repeat both the curve shape and the specific capacity of the first cycle. After the second discharge, a high reversible capacity of 173 and 101 mAh g^−1^ can be obtained for NVONBs and NVOP, respectively, which demonstrate that the layered structure NVO is suitable for Na ion insertion/extraction towards a promising cathode material for NIB. However, it should be noted that, compared to that of NVONBs, more obvious polarization exist for NVOP during the charge/discharge process, suggesting that the serious kinetic frustration of the NVOP, which might be resulted from the insufficient electronic and ionic conductivities. The combination of higher specific capacity and lower polarization demonstrates the advantage of nanobelts and single‐crystal structure of NVO.

Cycling stability of NVONBs is then investigated by galvanostatic charge and discharge technique between 1.5 and 3.8 V at a current density of 50 mA g^−1^. As shown in Figure 3b, the initial discharge capacity is 125 mAh g^−1^. Surprisingly, a high reversible capacity of 82 mAh g^−1^ can be obtained even after 190 cycles, corresponding to a small capacity loss of 0.21 mAh g^−1^ per cycle. The coulombic efficiency is more than 96% on average, indicating some irreversible reactions occur which might be related to the formation/decomposition of SEI.[[qv: 4b]],[[qv: 10c]] In sharp contrast, although a high initial reversible capacity of 105 mAh g^−1^ can be obtained for NVOP electrode, the cyclic stability is very poor. After only 20 cycles, the capacity decays sharply to a low capacity of 80.5 mAh g^−1^, showing much worse cycling stability than that of NVONB electrode. Furthermore, the NVONBs exhibit much better rate capability compared to the NVOP electrode operated at different current densities from 25 to 500 mA g^−1^. As shown in Figure 3c, even the current density increases 20 times to a high value of 500 mA g^−1^ (≈10 C, a rate of *n* C corresponds to a full discharge in 1/*n* h), the NVONBs can still retain over 35% (56.6 mAh g^−1^) of its initial reversible capacity (25 mA g^−1^: 161 mAh g^−1^). While even under a much lower current density of 200 mA g^−1^, the NVOP can maintain only 10% (10.3 mAh g^−1^) of its initial reversible capacity (103 mAh g^−1^). Importantly, when the current density turn back to the initial 25 mA g^−1^, the specific capacity of the NVONBs can still recover back to 103.6 mAh g^−1^, whereas that of NVOP is only 24.6 mAh g^−1^. The enhanced electrochemical performance of NVONBs might be attributed to the short electron transmission path of 1D nanobelts structure and the finite lattice defects of single‐crystal structure.^13^ In order to future understand the advantages of NVONBs, electrochemical impedance spectroscopy plots of NVONBs and NVOP electrodes at room temperature before and after ten cycles are showed in Figure 3d. It is found that the both NVONBs and NVOP electrodes hold similar shape of electrochemical impedance spectroscopy plots, composing of one semicircle component at high frequency range and followed by a linear component at the low frequency range. Obviously, the NVONBs electrode has a smaller semicircle than that of the NVOP, indicating a smaller electrochemical reaction resistance, which further indicates that the 1D nanobelts and the unique single‐crystal structure is beneficial to improve the conductivity of the NVO electrode and enhance the reaction kinetics, resulting in the better cycle and rate performance.

**Figure 4:**
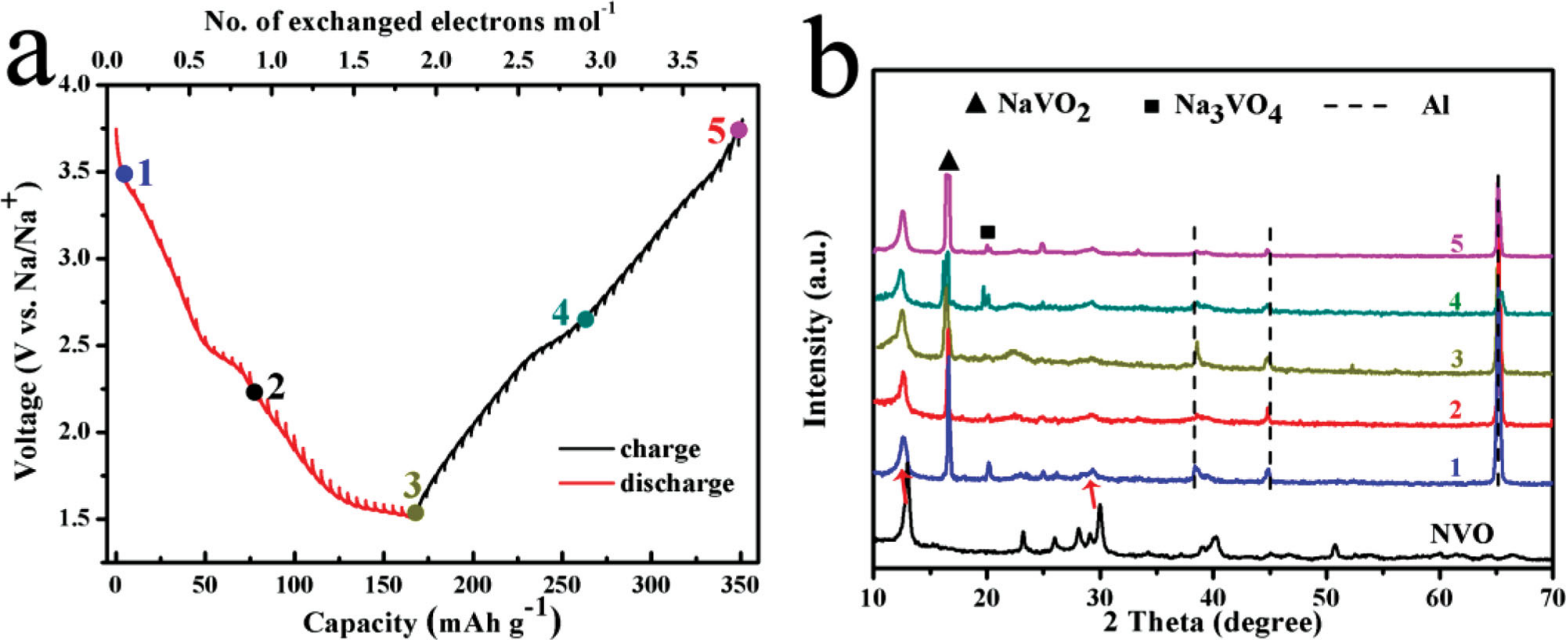
a) The GITT profiles of NVONBs versus Na measured at 5 mA g^−1^ in the second charging and discharging. b) Ex situ XRD patterns of NVONBs at different charge/discharge states after six cycles.

In order to further study the kinetic performance of Na ion in NVO electrode, the thermodynamic equilibrium potential of NVONB electrode is introduced. The NVONB battery is charged to 3.8 V versus Na before starting the discharge by using the galvanostatic intermittent titration technique (GITT) mode. **Figure** **4**a presents the quasi‐equilibrium redox potential of the NVONB electrode at a current density of 5 mA g^−1^. It is found that the shape of the GITT curve is very similar to the above obtained discharge‐charge curve under continuous charge and discharge (Figure 3a), indicating that even in the continuous discharge‐charge process, the NVONBs material is very close to the equilibrium state, which might be due to its good ionic and electronic conductivity. In addition, nearly 1.8 Na ions per formula unit could be inserted into NVONBs and then almost equal amount of Na ions extract from NVONBs during the following charge process. To further investigate the reversible structure changes of NVONBs upon Na^+^ insertion/deinsertion, we conducted ex situ XRD measurements on the electrodes at different charge/discharge states (Figure 4b), corresponding to the points 1–5 in Figure 4a. Due to the intercalation of Na ions, the peak positions of NVONBs shift to left a little. Although some new phases (NaVO_2_, Na_3_VO_4_, etc.) appeared, the main crystal structure of NVO can still maintain even after six cycles charged/discharged. It is worth noting that some weak peaks can also be found during charging/discharging, unfortunately, which cannot be indexed to any known phase according to the standard crystallographic database accurately. These results confirm the complexity of the electrochemical reactions of AVMs and the cycled product might be composed of Na–V–O and V–O matrixes.[[qv: 12a]],^14^

**Figure 5:**
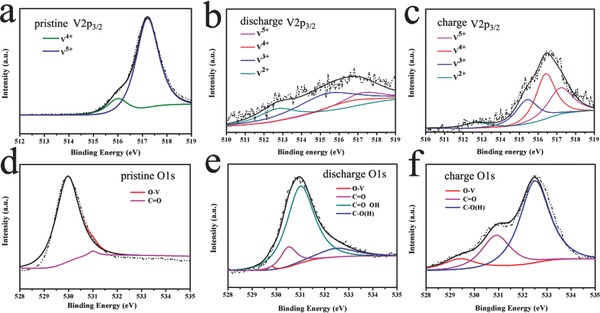
a–b) XPS spectra of V 2p_3/2_ core level regions and d–f) O 1s core level regions before and after six cycles of charged/discharged with Na ion.

To further investigate the phase changes of NVO upon Na insertion/extraction, X‐ray photoelectron spectroscopy (XPS) is employed on NVONB electrodes at different charge/discharge states. **Figure** **5**a–c show XPS spectra of NVONBs of in the V 2p_3/2_ core level regions before and after six cycles charged to 3.8 V and discharged to 1.5 V. Before electrochemical measurement, the peak at 517.2 eV is assigned to V^5+^ and another peak at 516.5 eV is assigned to V^4+^.^15^ Composition of the NVONB electrodes before the intercalation of Na^+^ shows 86% of total vanadium in V^5+^ and 14% in V^4+^. After six cycles, V 2p_3/2_ peak shape for the sample in the discharged state constitute 21.1% of V^5+^, 13% of V^4+^, 46.7% of V^3+^ (515.4 eV), and 19.2% of V^2+^ (512.7 eV), respectively;^16^ while in the charged state, it contains 29.3% of V^5+^, 39.4% of V^4+^, 24.5% of V^3+^, and 6.8% of V^2+^, respectively. According to the XPS spectra, vanadium can interchange from V^5+^ to V^2+^ during the process of intercalation and deintercalation of Na^+^ ion in the NVONBs and most of these changes are occurred between V^5+^ and V^3+^. Although some small amount of irreversible low state vanadium (V^2+^) exist in these complex electrochemical reactions, the reversible vanadium interchange dominates the Na ion insertion/extraction processes, which is consistent with the result of XRD in Figure 4b and previous reports.^14^ Figure 5d–f shows XPS spectra of O 1s regions before and after electrochemical reaction, respectively. In Figure 5d, the peaks of O 1s can be attributed to lattice oxygen O–V and C=O located at 530 and 530.5 eV.[[qv: 12b]],^15^ After six cycles (Figure 5e,f), several new peaks appeared. According to literature, the O 1s binding energy for C=O, OH, and C–O(H) are between 530.7–531.6 and 532.1–533.2 eV, respectively.[[qv: 12b]],^15^, ^17^ During charging/discharging process, the intensity of V–O changes obviously, which is due to the conservation of low entropic energy associated with ordering of intercalated atoms. It is worth noting that the C–O, OH, C–O(H) peaks enhanced significantly, the analyses of wide scan spectra (Figure S4, Supporting Information) for NVONBs and O 1s demonstrate the formation of a stable SEI layer and chemisorbed water, carbon dioxide molecules, or surface adsorption electrolyte molecules.[[qv: 12b]],^15^

The above obtained superior electrochemical performances of NVONBs electrode could be reasonably attributed to the follow reasons: First, the short transport length of 1D nanobelts makes full Na diffusion possible within a short diffusion time, at high charge/discharge current rates. Additionally, 1D nanobelts can also provide a higher electrode/electrolyte contact area that is beneficial to high current rate performance.^10^ Second, Na^+^ can be inserted into the layered structure of NVO, wherein the ample open space between the layers not only allows for easy diffusion of Na^+^ into the inner area of the NVO but also allows the electrochemical activity of NVO to be efficiently utilized and accommodated of the volume variation during the Na^+^ uptake and release process, which could restrain the pulverization.^10^,[[qv: 12b]],^15^, ^18^ As shown in Figure S5, Supporting Information, even after 1000 cycles, the NVONBs can also show more than 42 and 30 mAh g^−1^ at 200 and 400 mA g^−1^, respectively. However, there is only 39 mAh g^−1^ at 200 mA g^−1^ after 300 cycles. What is important, the morphology of NVONBs is kept as well as it is before Na^+^ insertion/deinsertion. As shown in Figure S6, Supporting Information, even after 1000 cycles, this electrodes are still dominated by nanobelt structures, however, even after 70 cycles, the morphology of NVOP cannot be kept (Figure S7, Supporting Information). Furthermore, the possible reason of the excellent performance of NVONBs is that in the single‐crystal Na‐ion does not need to across grain boundary and overcome the barrier of defects, so the Na‐ion diffusion coefficient in this single‐crystal NVONBs should be higher than the material composed by aggregate of small particles.^13^ It is suggesting that Na^+^ insertion/extraction with in this compound is quite reversible, this excellent stability could due to the reversibility phase transition during repeated charging/discharging process, and highlight the feasibility and strength of the concept nanoscale, single‐crystal and layered structure for improving NIBs performance.

## 3 Conclusions

In summary, pure single‐crystal NVONBs are successfully synthesized and its layer structure is demonstrated by Rietveld refinement method for the first time. Unexpectedly, when applied as novel cathode materials in NIBs, the obtained NVONBs show superior electrochemical performances, including high reversible capacity up to 173 mAh g^−1^, good cycling stability over 190 cycles, and good rate capability, which might be attributed to the layered and 1D pure single‐crystal structure of NVO. Ex situ XRD and XPS techniques are used to discuss the phase transformation along the discharge/charge process. The obtained results clearly demonstrate the promising potential of NVONBs as superior cathode materials for NIBs, which might help bring the low‐cost room‐temperature NIBs a step closer to a sustainable large‐scale electric energy storage system.

## Supporting information

As a service to our authors and readers, this journal provides supporting information supplied by the authors. Such materials are peer reviewed and may be re‐organized for online delivery, but are not copy‐edited or typeset. Technical support issues arising from supporting information (other than missing files) should be addressed to the authors.

Supplementary
