# Supplementary material for: Pure Single‐Crystalline Na1.1V3O7.9 Nanobelts as Superior Cathode Materials for Rechargeable Sodium‐Ion Batteries
Source: Adv Sci (Weinh). 2015 Feb 17;2(3):1400018. doi: 10.1002/advs.201400018 (PMC5115286; doi:10.1002/advs.201400018)
Supplement: Supplementary file 1 — Supplementary [file ADVS-2-0e-s001.pdf]

Copyright WILEY-VCH Verlag GmbH & Co. KGaA, 69469 Weinheim, Germany, 2015.

ADVANCED SCIENCE
Open Access

Supporting Information

for *Adv. Sci.*, DOI: 10.1002/advs.201400018

Pure Single-Crystalline $Na_{1.1}V_3O_{7.9}$ Nanobelts as Superior Cathode Materials for Rechargeable Sodium-Ion Batteries

*Shuang Yuan, Yong-Bing Liu, Dan Xu, De-Long Ma, Sai Wang, Xiao-Hong Yang, Zhan-Yi Cao, and Xin-Bo Zhang**

Supporting information

Pure Single-Crystalline $Na_{1.1}V_3O_{7.9}$ Nanobelts as Superior Cathode Materials for Rechargeable Sodium-ion Batteries

Shuang Yuan, Yong-bing Liu, Dan Xu, De-long Ma, Wang-dong Lu, Sai Wang, Xiao-hong Yang, Zhan-yi Cao and Xin-bo Zhang*

[*] Mr. Shuang Yuan, Ms. Dan Xu, De-long Ma, Ms. Wang-dong Lu, Mr. Sai Wang, Prof. Dr. Xin-bo Zhang
State Key Laboratory of Rare Earth Resource Utilization, Changchun Institute of Applied Chemistry, Chinese Academy of Sciences
Changchun, 130022, China
E-mail: xbzhang@ciac.ac.cn
Group webpage: http://energy.ciac.jl.cn
Mr. Shuang Yuan, Mr. De-long Ma, Mr. Sai Wang, Prof. Dr. Yong-bing Liu, Prof. Dr. Xiao-hong Yang, Prof. Dr. Zhan-yi Cao
Key Laboratory of Automobile Materials, Ministry of Education and School of Materials Science and Engineering, Jilin University, Changchun 130012, China
Ms. Wang-dong Lu
University of Chinese Academy of Sciences
Beijing, 100049 P. R. China

Experimental Section

Synthesis of single-crystalline $Na_{1.1}V_3O_{7.9}$ nanobelts: All the reagents used in the experiment were of analytical grade purity and were used as received. The starting materials, vanadium oxide and sodium hydroxide were of analytically pure grade. Firstly, 10 mmol V_2O_5 (Beijing Chemical Works) and 10 mmol NaOH (Beijing Chemical Works) were dissolved in deionized water successively. The mixed solution was then transferred to 50 ml Teflon lined stainless steel autoclave. The autoclave was sealed and heated at 180 ℃ for 36 h and then cooled to room temperature naturally. The obtained precipitates were filtered, washed with deionized water at least three times to obtain pure $Na_2V_6O_{16}$ nanobelts. Finally, the $Na_2V_6O_{16}$ nanobelts were dried at 205 ℃ for 6 hours to obtain homogeneous single-crystalline $Na_{1.1}V_3O_{7.9}$ nanobelts.

Synthesis of irregularly-shaped $Na_{1.1}V_3O_{7.9}$: First, irregularly-shaped V_2O_5 particles power was obtained as follow:[S1] V_2O_5 (1.2 g) and $H_2C_2O_4$ (3.0 g) in a stoichiometric ratio of 1:5 were added to 30 ml distilled water under active stirring at room temperature until the color of the solution changed from yellow to blue. The solution was dried at 80 ℃ to produce the precursor and it was then calcined at 400 ℃ in air for 2 h. Then irregularly-shaped $Na_{1.1}V_3O_{7.9}$ nanoparticles (NVOP) were obtained as mentioned above.

Material Characterization: Powder X-ray diffraction (XRD) measurement was performed on a Bruker D8 Focus Powder X-ray diffractometer using Cu Kα (λ = 0.15405 nm) radiation (40 Kv, 250 Ma). The phase compositions of the samples were determined through refining the diffraction patterns with GSAS program based on the

Rietveld method.[S2] Scanning electron microscopy (SEM) was performed on a field emission Hitachi S-4800 instrument, operating at an accelerating voltage of 10 Kv. Transmission electron microscope (TEM) was performed using a FEI Tecnai G_2S-Twin instrument with a field emission gun operating at 200 Kv. XPS spectra were obtained with an ESCALAB MK II X-ray photoelectron spectrometer using an Al Kα source. Fourier transform infrared (FTIR) spectra were recorded on a Bruker Vertex70 spectrometer.

Electrochemical Measurement: The electrodes were prepared by mixing active material (80 wt%), acetylene black (10 wt%), and carboxyl methyl cellulose (CMC, 10 wt%) in deionized water. After the slurries were uniformly spread onto a aluminum foil, the electrodes were dried at 80 ℃ in vacuum for 12 h. The electrodes were then pressed and cut into disks before transferring into an argon-filled glove box. The average loading of active material is ca. 0.8 mg. Coin cells (CR2025) were assembled in the laboratory by using Na metal as counter electrode, Celgard 2400 membrane as separator, and $NaPF_6$ (1 M) in ethylene carbonate/diethyl carbonate (EC/DEC, 1:1 v/v) as electrolyte. Galvanostatic charge–discharge tests were carried out on a Land Battery Measurement System (Land, PR China). Nyquist plots were performed by using a VMP3 Electrochemical Workstation (Bio-logic Inc.).

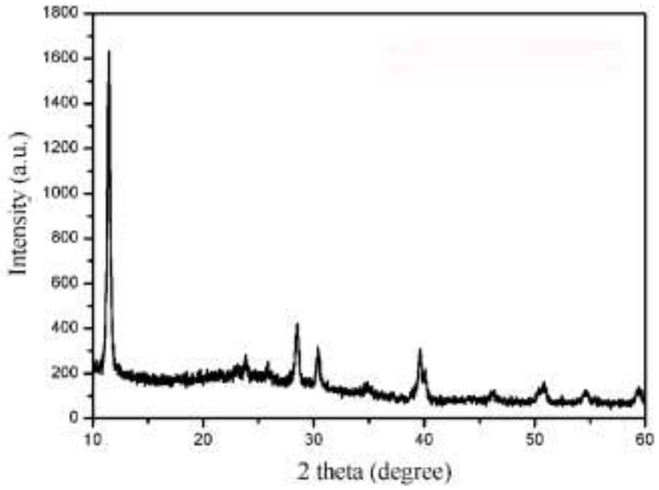

Figure S1. XRD pattern of $Na_2V_6O_{16}$ nanobelts.

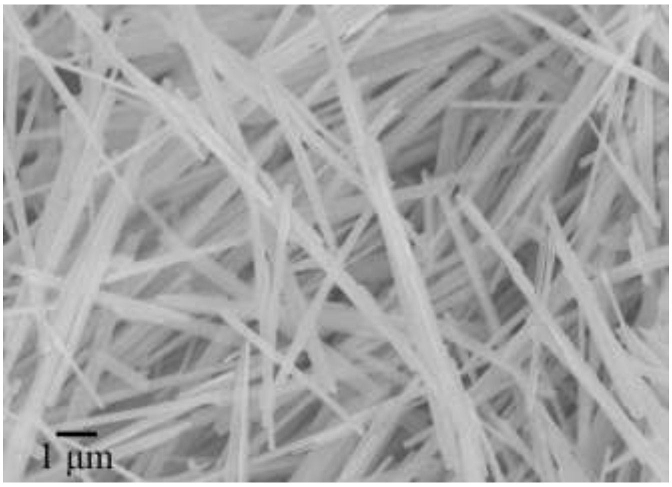

Figure S2. FESEM image of $Na_2V_6O_{16}$ nanobelts.

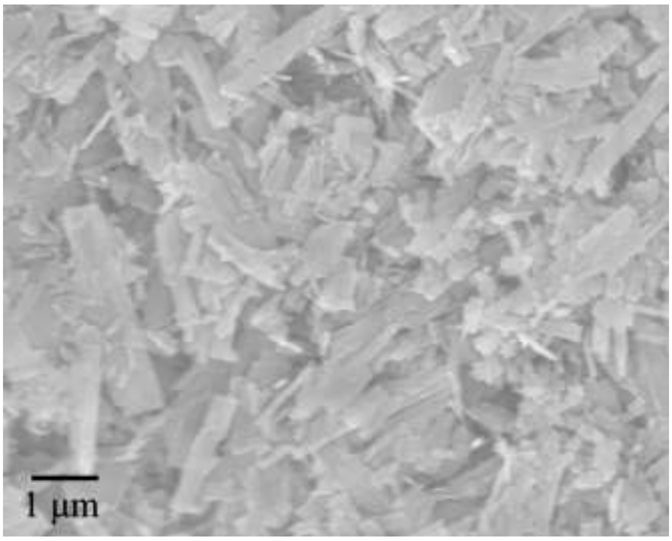

Figure S3. FESEM image of irregularly-shaped NVO powder (NVOP).

Table S1. Comparison of electrochemical performance of different cathode materials for NIBs.

Cathode Materials	Capacity (mAh g^{-1})	Rate Capability (mAh g^{-1})	Energy Density (Wh kg^{-1})	Cycle Stability	Reference
$NaVOPO_4/C$	90	90 (10 mA g^{-1})	234	30	S4
$Na_{0.67}Mn_{1-x}Mg_xO_2$	175	175 (12 mA g^{-1})	420	25	S5
$Na_{2.24}FePO_4CO_3$	125	58 (200 mA g^{-1})	312	40	S6
$Na_2V_3(PO_4)_2F_3$	115	110 (0.091C)	442	50	S7
$Na_3MnPO_4CO_3$	125	60 (1C)	390	15	S8
$Na_{2/3}Co_{2/3}Mn_{2/9}Ni_{1/9}O_2$	105	67 (2C)	336	90	S9
Nickel ferricyanide	60	—	214	200	S10
$NaVO_3$	175	—	280	5	S11
NVOP	103	10 (200 mA g^{-1})	257	20	This work
NVONBs	173	57 (500 mA g^{-1})	432	190	This work

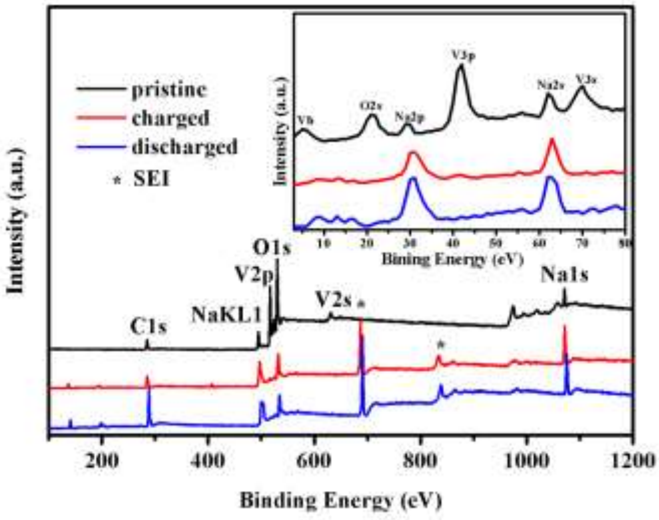

Figure S4. Wide scan spectra for the cleaved of as-prepared NVONBs. The inset shows the binding energy signal from 0 to 80 eV.

Figure S5 shows wide scan spectra for the cleaved of as-prepared NVONBs before and after electrochemical reaction. The inset shows the binding energy signal from 0 to 80 eV. After electrochemical reaction, C 1s, Na KL1 and Na 1s peaks increase obviously, and the peaks of V changed a lot. These changes are corresponding to the valence changed during the electrochemical reaction. There are also some peaks correspond to F, it was due to electrolyte decomposition and is likely to form a stable SEI.[S3]

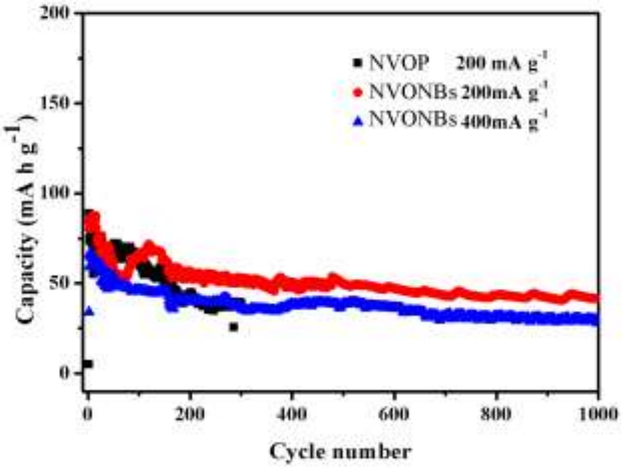

Figure S5. Discharge profiles of NVOP at 200 mA g^{-1} after 300 cycles and NVONBs at 200/400 mA g^{-1} after 1000 cycles.

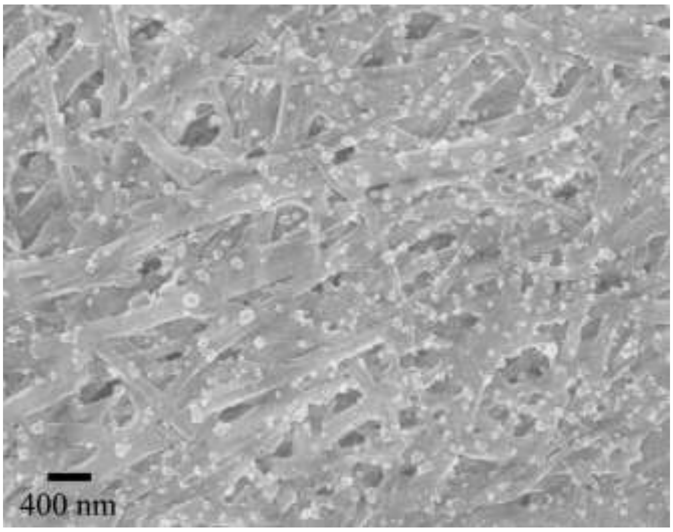

Figure S6. FESEM image of NVONBs after 1000 cycles.

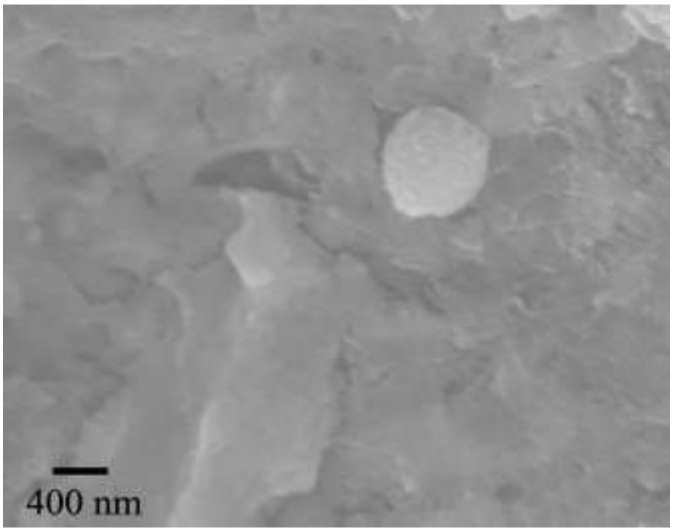

Figure S7. FESEM image of NVOP after 70 cycles.

References

[S1] A.Q. Pan, J. G. Zhang, Z. Nie, G. Z. Cao, B. W. Arey, G. Li, S. Q. Liang, J. Liu, *J. Mater. Chem.* **2010**, *20*, 9193.

[S2] B. H. Toby, EXPGUI, a graphical user interface for GSAS, *J. Appl. Cryst.* **2001**, *34*, 210.

[S3] K. Ha, S. Woo, D. Mok, N. Choi, Y. Park, S. M. Oh, Y. Kim, J. Kim, J. Lee, L. F. Nazar, K. Lee, *Adv. Energy Mater.* **2013**, *3*, 770.

[S4] J. Song , M.Xu, L. Wang, J. B. Goodenough, *ChemComm.* **2013**, *49*, 5280.

[S5] J. Billaud, G. Singh, A. R.Armstrong, E. Gonzalo, V. Roddatis, M. Armand, T. Rojo, P. G. Bruce, *Energy Environ. Sci.* **2014**, DOI: 10.1039/C4EE00465E.

[S6] W. Huang, J. Zhou, B. Li, J. Ma, S. Tao, D. Xia, W. Chu, Z. Wu, *Sci. Rep.* **2014**, *4*, 4188.

[S7] W. Song, X. Ji, Z. Wu, Y. Yang, Z. Zhou, F. Li, Q. Chen, C. E. Banks, *J. Power Soucres* **2014**, *256*, 258.

[S8] H. Chen, Q. Hao, O. Zivkovic, G. Hautier, L. Du, Y. Tang, Y. Hu, X. Ma, C. P. Grey, G. Ceder, *Chem. Mater.* **2014**, dx.doi.org/10.1021/cm400805q.

[S9] S. Doubaji, M. Valvo, I. Saadoune, M. Dahbi, K. Edstrom, *J. Power Soucres* **2014**, *26*, 275.

[10] Y. You, X. L. Wu, Y. X. Yin, Y. G. Guo, *J. Mater. Chem. A* **2013**, *1*, 14061.

[S11] G. Venkatesh, V. Pralong, O.I. Lebedev, V. Caignaert, P. Bazin, B. Raveau, *Electrochem. Commun.* **2014**, *40*, 100.
